# Naturalistic Stimulus Structure Determines the Integration of Audiovisual Looming Signals in Binocular Rivalry

**DOI:** 10.1371/journal.pone.0070710

**Published:** 2013-08-27

**Authors:** Verena Conrad, Mario Kleiner, Andreas Bartels, Jessica Hartcher O'Brien, Heinrich H. Bülthoff, Uta Noppeney

**Affiliations:** 1 Max Planck Institute for Biological Cybernetics, Tübingen, Germany; 2 Werner Reichardt Centre for Integrative Neurosciences, Tübingen, Germany; 3 Department of Brain and Cognitive Engineering, Korea University, Anam-dong, Seongbuk-gu, Seoul, Korea; 4 Computational Neuroscience and Cognitive Robotics Centre, University of Birmingham, Birmingham, United Kingdom; University of California Davis, United States of America

## Abstract

Rapid integration of biologically relevant information is crucial for the survival of an organism. Most prominently, humans should be biased to attend and respond to looming stimuli that signal approaching danger (e.g. predator) and hence require rapid action. This psychophysics study used binocular rivalry to investigate the perceptual advantage of looming (relative to receding) visual signals (i.e. looming bias) and how this bias can be influenced by concurrent auditory looming/receding stimuli and the statistical structure of the auditory and visual signals.

Subjects were dichoptically presented with looming/receding visual stimuli that were paired with looming or receding sounds. The visual signals conformed to two different statistical structures: (1) a ‘simple’ random-dot kinematogram showing a starfield and (2) a “naturalistic” visual Shepard stimulus. Likewise, the looming/receding sound was (1) a simple amplitude- and frequency-modulated (AM-FM) tone or (2) a complex Shepard tone. Our results show that the perceptual looming bias (i.e. the increase in dominance times for looming versus receding percepts) is amplified by looming sounds, yet reduced and even converted into a receding bias by receding sounds. Moreover, the influence of looming/receding sounds on the visual looming bias depends on the statistical structure of both the visual and auditory signals. It is enhanced when audiovisual signals are Shepard stimuli.

In conclusion, visual perception prioritizes processing of biologically significant looming stimuli especially when paired with looming auditory signals. Critically, these audiovisual interactions are amplified for statistically complex signals that are more naturalistic and known to engage neural processing at multiple levels of the cortical hierarchy.

## Introduction

Perception is intimately linked to action. It should provide an organism with representations that enable effective interactions with the environment. Perceptual representations may therefore not always reflect the statistical structure of the world in a veridical fashion but place greater emphasis on stimuli that are biologically relevant and critical for an organism's survival. Thus, the brain may be biased to attend to and encode looming stimuli that signal approaching danger and hence require rapid ‘flight or fight’ action [1]–[3]. One may even speculate whether perceptual biases such as the looming bias may have become hardwired in the neural architecture as a consequence of selection pressures that are active in the course of evolution.

Indeed, evidence from psychophysics and neurophysiology has accumulated showing a perceptual asymmetry for looming relative to receding signals [4]–[9]. For instance, humans respond faster to looming sounds and underestimate their time of arrival [5]. Looming stimuli also induce greater phasic alertness as indexed by increased skin conductance responses [9]. Finally, when presented with signals in audition and vision, humans show a greater behavioural benefit for integrating looming relative to static signals [10], [11], cf. [12] for a developmental perspective.

This looming/receding asymmetry has also been observed at the neural level with responses in the lateral belt area of the macaque auditory cortex [13] and the superior temporal sulcus [14] being increased for looming relative to receding stimuli. Likewise, multisensory neurons in the ventral intraparietal area and precentral gyrus responded primarily to visual, tactile and auditory looming stimuli as a putative mechanism for defense of the body surface [15].

Perceptual advantages and asymmetries are most pronounced in multistable perception, when the brain alternates between multiple similarly likely perceptual interpretations. Indeed, in binocular rivalry the dominance times of the looming percept exceeded those of the competing receding percept indicating that the looming signals are preferentially processed and prioritized to access human awareness [16]. Moreover, concurrently presented looming sounds amplify this looming bias by prolonging the dominance times of the visual looming percept and abbreviating those of the receding percept. Yet surprisingly, receding sounds were not able to convert the looming bias into a receding bias – a finding possibly pointing towards stronger multisensory interactions for looming than receding concentric gratings [17]. Collectively, these findings demonstrate that looming signals are integrated across multiple senses into perceptual, attentional and decisional advantages for looming signals [17], [18].

The current study investigated how auditory and visual looming signals interact during binocular rivalry. First, we revisited the question whether receding auditory signals can invert a looming bias into a receding bias. Second, we examined whether the visual looming bias per se as well as the influence of a looming sound on the visual looming bias depends on the statistical structure of the auditory and visual signals.

To address these questions, in a binocular rivalry paradigm we presented subjects dichoptically with looming/receding visual stimuli that were paired with looming or receding sounds. The visual signals conformed to two different statistical structures: (1) a ‘simple’ random-dot kinematogram showing a starfield and (2) the visual Shepard stimulus that follows the characteristic 1/f amplitude spectrum of natural image scenes. Likewise, the looming/receding sound was (1) a simple AM-FM modulated tone or (2) a complex Shepard tone [19]–[22].

We expected complex stimuli to enforce stronger audiovisual interactions by providing perceptually more convincing and immersive cues that inform the brain of potential danger or collision. Furthermore, the brain may have developed perceptual and multisensory binding mechanisms finetuned to the natural statistics of auditory and visual signals [23]–[25]. Finally, complex stimuli may facilitate audiovisual interactions, as they are processed at multiple levels of the cortical hierarchy [26].

## Materials and Methods

### Ethics statement

This study was conducted in accordance with the declaration of Helsinki, and had ethical approval from the local ethics committee of the University of Tübingen. All participants provided written informed consent and received 8 € per hour in return.

### Participants

Sixteen observers participated in the study (9 females; two left-handed). All observers had normal or corrected-to-normal vision and were aged 24–38 years (mean: 30.19; SD: 4.69). All observers were naïve with regard to the purpose of this study except for one observer (VC) who is an author of the manuscript. No participant reported any hearing deficits.

### Stimuli

#### Visual stimuli

**Figure 1:**
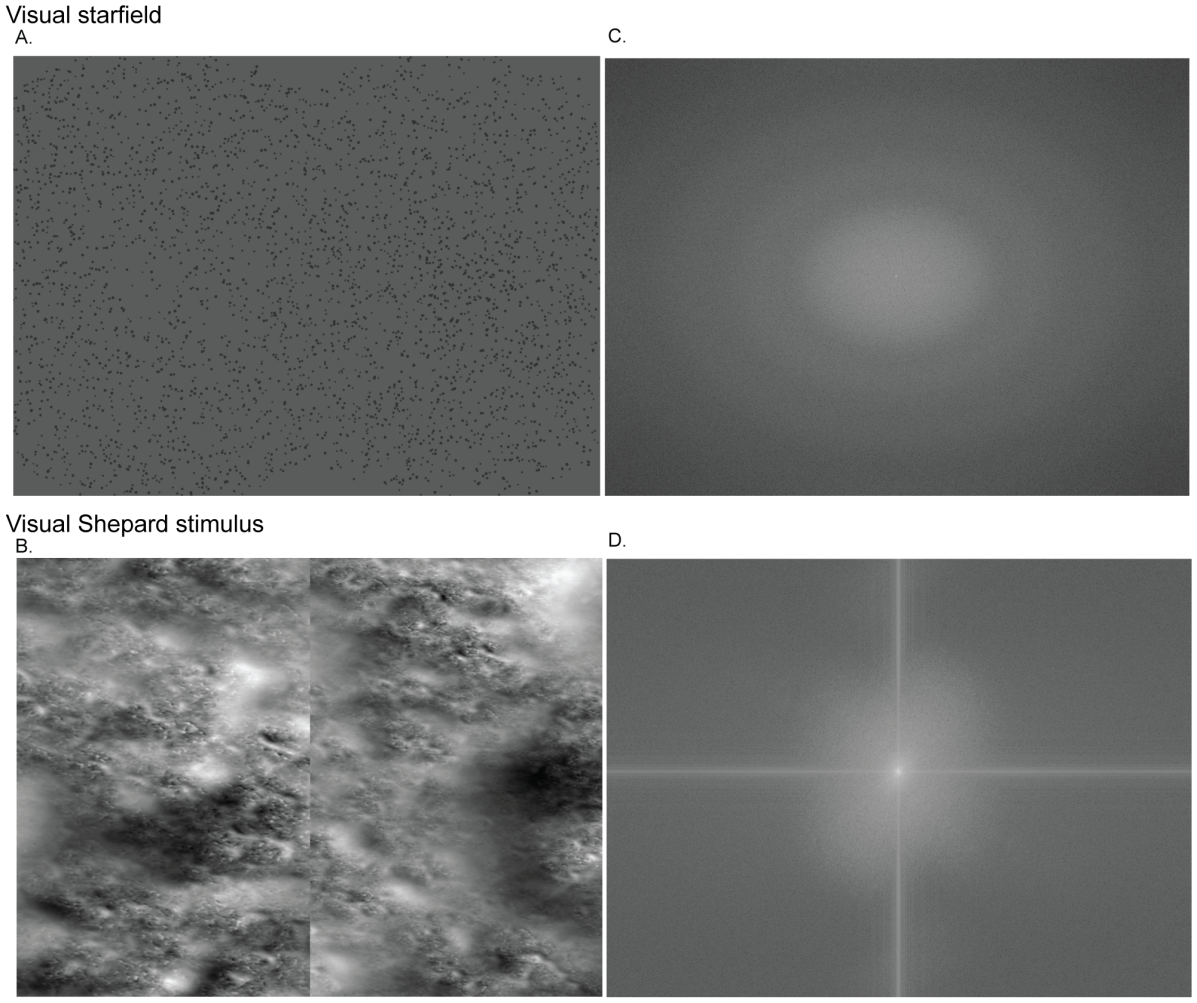
Random dot visual starfield and visual Shepard stimulus in image space and in Fourier space (A–D). Figure 1 shows the random dot visual starfield (A) and visual Shepard stimulus (B) in image space. On the right the same images are shown in Fourier space (C, D). Here, amplitudes of frequencies are coded as brightness. Lowest spatial frequencies are represented in the middle of each image, with increasing frequencies towards the image border. The visual Shepard stimulus is a multi-scale texture, consisting of five layers of semi-transparent smoothed random noise at different scales. The layers are rendered smallest scale first, towards larger scale. This results in a texture with an amplitude spectrum close to the natural 1/f – the spectral signature of natural scenes. The lowest spatial frequencies have the highest amplitude, suggesting that the low frequencies contain more image information than higher ones. (For a detailed description of the stimulus see [27]).

Two types of visual stimuli were used to generate looming and receding motion: a starfield implemented in a random-dot kinematogram (RDK) and the visual Shepard stimulus (see Figure 1). The two types of stimuli were matched for root mean square contrast (RMS) and radial velocity, yet differed in their spectral energy content (Figure S1). Visual stimuli were presented dichoptically using a stereoscope consisting of two CRT monitors viewed via angled, silver-coated mirrors. The monitors were independently linearized, and run with a resolution of 1024×768 for our stimuli. Visual stimuli were presented on a grey, uniform background with a mean luminance of 20.34 cd/m^2^. The viewing distance was 118 cm. Both types of psychophysical stimuli were generated on a standard PC running Windows XP using the Psychtoolbox Version [27], [28] (http://psychtoolbox.org) in Matlab 7 (Mathworks, Nantucket, Massachusetts).

#### Visual starfield

The looming/receding starfield was a limited lifetime random dot kinematogram (RDK) of contracting (i.e. receding) or expanding (i.e. looming) radial motion at a constant speed of 1.5 deg/sec and 100% motion coherence. The randomly positioned dots (Ø 0.1° visual angle) were isoluminant dark grey on a grey background. The RDKs were presented foveally, together with an isoluminant fixation spot of 0.5° and framed by an isoluminant grey square aperture of 3° in diameter to aid binocular fusion. Mean luminance of the visual starfield was 19.02 cd/m^2^.

#### Shepard image sequence

The looming/receding Shepard image sequence is the visual equivalent of the Shepard tone illusion [29]. It is created using multi-layer spectral texturing (for a detailed description see [30], [31]; for a demonstration see Psychtoolbox/PsychDemos/OpenGL4MatlabDemos/ShepardZoomDemo). Spectral texturing models the statistical relationships between bands of the texture's spatial spectrum resulting in realistic textures with an overall amplitude spectrum close to 1/f mimicking the amplitude spectrum of natural images [32]. The textures were presented foveally at a viewing distance of 118 cm, contained an isoluminant grey fixation spot of 0.5° of visual angle and framed by an isoluminant grey square aperture of 3° in diameter to aid binocular fusion. Mean luminance of the textures was 22.8 cd/m^2^. The scene was rendered in real-time using OpenGL functions via Psychtoolbox in Matlab on a standard PC with a standard graphics card. Observers interpreted the stimuli as rocks or heavy clouds coming towards them or moving away.

#### Auditory stimuli

**Figure 2:**
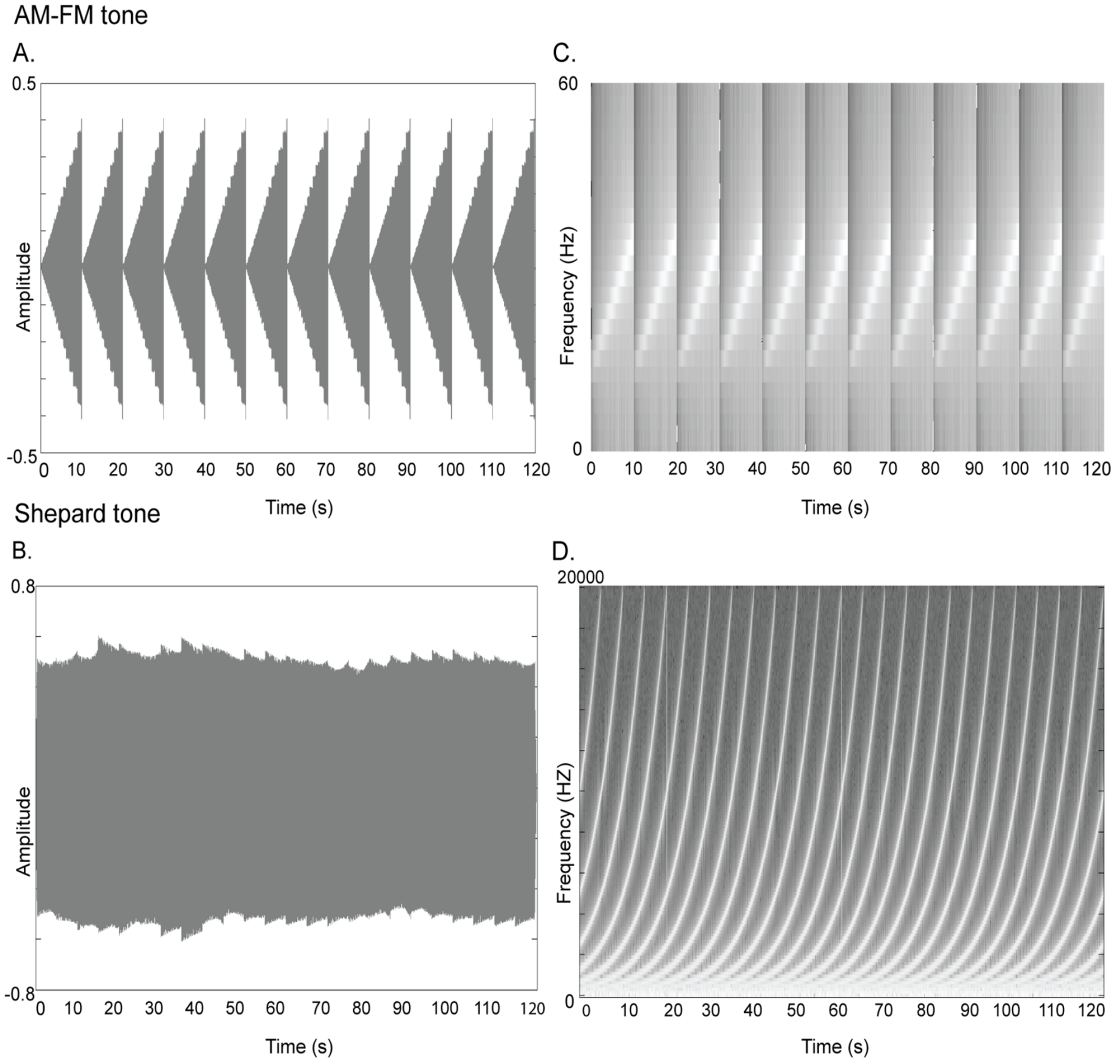
Sound waveforms and time-frequency representations (A–D). Sound waveforms (left) and time-frequency representations (right) of the amplitude and frequency modulated (AM-FM) tone (top: A, C) and the Shepard tone (bottom: B, D).

The looming/receding sounds were (1) an AM-FM modulated tone or (2) a complex Shepard tone (see Figure 2). Both types of auditory stimuli were digitized at a sampling rate of 44.1 kHz via a standard sound card and delivered binaurally through a pair of headphones (Sennheiser HD 201) at an average of 70 dB SPL as measured with a SPL meter directly placed on the headphones (Brüel & Kjaer, Norcross, GA). Figure 2C and 2D show the difference in frequency spectra for the AM-FM tone and the Shepard tone. Auditory stimuli were edited in Adobe Audition (Adobe Systems, San Jose CA) and Matlab.

#### Amplitude- and Frequency-modulated tones (AM-FM)

The auditory looming (or receding) stimulus was generated by concatenating 12 identical segments of AM-FM modulated tones of 10 s duration. In each looming segment, the frequency increased from 200 Hz to 380 Hz and the amplitude from 65 db to 75 db. Conversely, in each receding segment, the frequency dereased from 380 Hz to 200 Hz and the amplitude from 75 db to 65 db. This created the impression of a sound source constantly approaching (or departing) in depth. The sound was created using Open AL to control properties of the auditory motion stimulus such as position, velocity and motion direction (for further reference for the use of OpenAL in psychophysical studies, see [28]).

#### Shepard tones

Auditory stimuli were continuous Shepard scales that consist of a superposition of sine waves separated by octaves and rising in frequency (for a detailed description see [29]). If sine waves reach the upper boundary of the perceptible frequency range, they are faded out and replaced by new sine waves at the lower boundary of the spectrum. Thereby the Shepard scale repeats itself in cycles. This creates the auditory illusion of a tone that continually ascends or descends in pitch, even though it does not truly get higher over time. We will refer to the ascending/rising complex Shepard tone as the “looming” signal and the descending/falling complex Shepard tone as the “receding” signal. Shepard tones are immersive complex tones.

### Experimental paradigm

**Figure 3:**
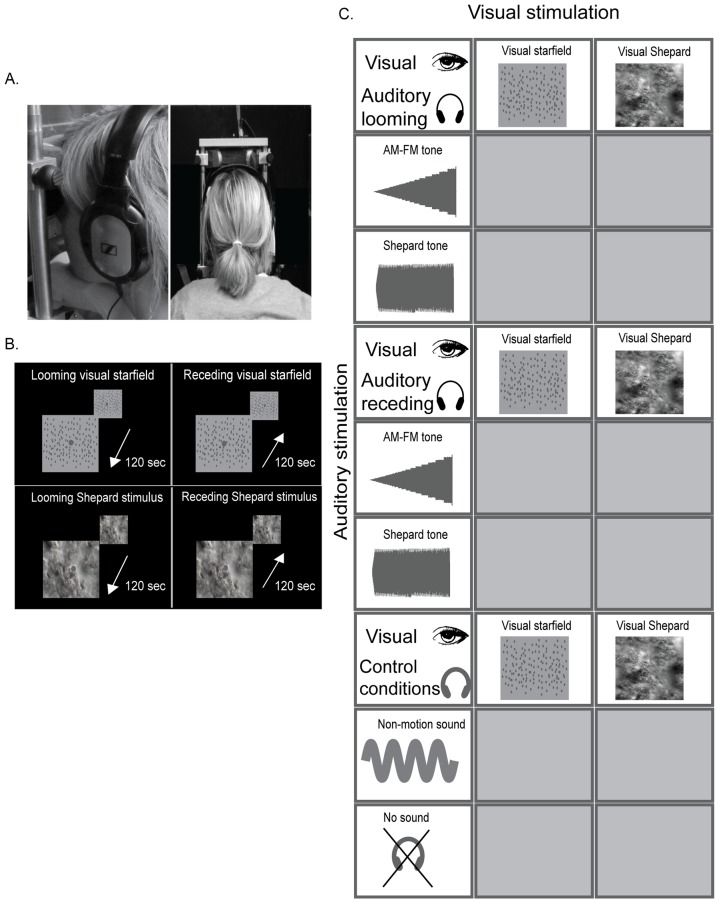
Experimental paradigm and stimuli (A–C). In a binocular rivalry paradigm, observers' eyes were presented concurrently with looming and receding motion. The visual stimulus was either a random dot visual starfield or a complex Shepard image sequence. The auditory stimulus was absent, a static sound or a looming or receding sound that was produced using an amplitude- and frequency modulated simple sound or a complex Shepard sound. Thus, the directional sound was congurent with the visual stimulus presented to one eye and inconsistent with that presented to the other eye. In the two control conditions either non-motion (stationary) sound or no sound was presented. (The subject of the photograph has given written informed consent, as outlined in the PLOS consent form, to publication of her photograph.).

In a binocular rivalry paradigm, subjects were dichoptically presented with looming/receding visual stimuli that were paired with looming or receding sounds (see Figure 3). Subjects continuously reported their visual percept. The visual signals were of two different statistical structures: (1) a ‘simple’ random-dot kinematogram showing a starfield or (2) a complex visual Shepard stimulus. Likewise, the looming/receding sounds were (1) a simple AM-FM tone or (2) a complex Shepard tone.

Hence, the 2×2×2 factorial design manipulated: (1) direction of the sound: looming vs. receding sound, (2) statistical structure of the visual stimulus: simple starfield vs Shepard image sequence, (3) the statistical structure of auditory stimulus: AM-FM tone vs. Shepard tone. In addition, we included conditions with a static sound and without sound for visual complex and simple signals. In the static sound condition participants were presented with a tone of 200 Hz (i.e. matched to the carrier frequency of the AM-FM tone). In the no-sound condition visual stimuli were presented in the absence of auditory inputs. The unisensory condition served as a baseline for identifying the looming bias under purely visual stimulation. This enabled us to test for an overall influence of auditory input on the temporal dynamics of binocular rivalry.

Subjects reported their visual percept – either looming or receding motion - by holding down one of two buttons and indicated mixed or indeterminate percepts by pressing neither button. They were instructed not to attend to the concurrent auditory input and to fixate throughout the duration of stimulus presentation.

Each experimental block lasted 120 seconds and was preceded and followed by a 2s-fixation period. In total, observers viewed two 120-s blocks of each of the conditions of rivalry presentations yielding 24 * 120-s blocks performed by each subject. The order of conditions was randomized and counterbalanced across subjects.

## Results

The analysis focused on the looming bias (i.e. the difference in perceptual dominance between looming and receding percepts). First, we investigated whether the looming bias per se depends on the statistical structure of the signals. Second, we examined the effect of a looming and receding sound on the visual looming bias. Third, we investigated how this auditory influence depends on the statistical structure of the visual and auditory stimuli.

**Figure 4:**
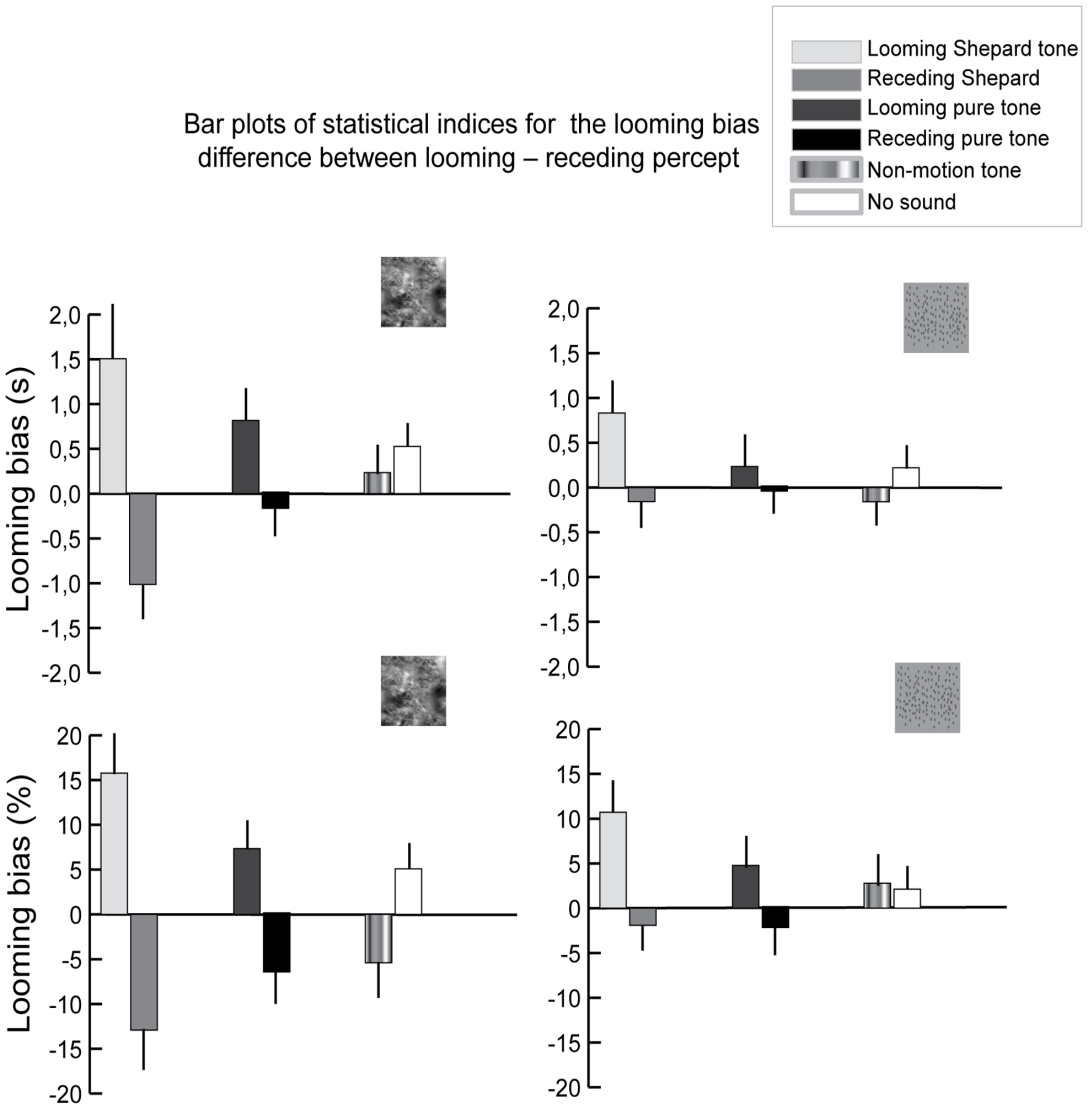
Bar plots of looming bias for mean dominance time (s, top) and % cumulative dominance time (bottom, across subjects mean+SE). Left: Visual Shepard stimulus; Right: Visual starfield. The type of auditory stimulation is colour coded (see legend). Please note that the looming bias differs for mean dominance times (in seconds) and % cumulative dominance times (i.e. fraction of total dominance duration for looming or receding percept and the total duration of presentation time) because of additional piecemeal periods.

To address these questions, we calculated the looming bias for mean dominance durations in seconds and % cumulative dominance time (i.e. total dominance duration for the looming percept/total duration of presentation time; n.b. as the total duration also includes piecemeal phases the % cumulative dominance times for looming and receding percepts do not sum to 100%) (see Figure 4). The looming bias for mean dominance durations and percent cumulative dominance were entered into separate 2×2×2 Repeated Measures ANOVAs with the factors (1) Visual statistical structure (visual Shepard stimulus or visual RDK starfield), (2) Auditory statistical structure (Shepard tone or tone) and (3) Sound direction (looming or receding). In the following, we report the main effects and interaction effects for each of the factors (see Table 1 and Table S1):

**Table 1:** Results of the 2×2×2 Repeated Measures ANOVAs with the factors (1) visual statistical structure (Shepard or starfield), (2) auditory statistical structure (Shepard tone or AM-FM tone) and (3) sound direction looming or receding) for mean dominance duration (s, left) and % cumulative dominance time (%, right) (Huynh-Feldt corrected).

Measures	Mean dominance duration in s	Dominance %
Visual statistical structure	F(1.00, 15.00) = 0.52, p>0.05	F(1.00, 15.00) = 0.59, p>0.05
Auditory statistical structure	F(1.00, 15.00) = 0.40, p>0.05	F(1.00, 15.00) = 2.24, p>0.05
Sound direction	F(1.00, 15.00) = 8.92, p<0.01	F(1.00, 15.00) = 11.96, p<0.01
Visual statistical structure×Auditory statistical structure	F(1.00, 15.00) = 0.24, p>0.05	F(1.00, 15.00) = 0.16, p>0.05
Sound direction×Visual statistical structure	F(1.00, 15.00) = 2.97, p = 0.1	F(1.00, 15.00) = 4.75, p<0.05
Sound direction×Auditory statistical structure	F(1.00, 15.00) = 7.13, p<0.05	F(1.00, 15.00) = 9.02, p<0.01
Sound direction×Visual statistical structure×Auditory statistical structure	F(1.00, 15.00) = 1.02, p>0.05	F(1.00, 15.00) = 3.48, p = 0.08

### Main effect of Visual statistical structure

There was no main effect of visual statistical structure. Hence, the looming bias was observed irrespective of the statistical structure of the visual stimuli (i.e. it was comparable for the visual Shepard stimulus and the visual starfield).

### Main effect of Auditory statistical structure

Likewise, the looming bias was not affected by auditory statistical structure, but commonly observed for a complex Shepard tone and an AM-FM tone.

### Main effect of Sound direction

We observed a significant main effect of sound direction. A looming sound amplified the looming bias, while a receding sound reduced it and even converted it into a receding bias.

### Interaction between Sound direction and Visual statistical structure

The significant interaction between sound direction and visual statistical structure demonstrated that the complexity of the visual stimulus modulates the influence of a looming or receding sound on the looming bias. The influence of a looming or receding sound was more pronounced for the complex visual Shepard stimulus.

### Interaction between Sound direction and Auditory statistical structure

Likewise, the significant interaction between sound direction and auditory statistical structure demonstrated the influence of a looming or receding sound on the looming bias was enhanced for complex Shepard sounds.

### 3-way interaction

We observed a non-significant trend for a 3-way interaction between sound direction, visual statistical structure and auditory statistical structure. In other words, the modulatory effect of a looming/receding sound on the looming bias was influenced in an interactive fashion by the statistical structure of the visual stimuli and the complexity of the auditory stimuli.

Collectively, these results demonstrate that the visual looming bias per se does not depend on the statistical structure of the auditory or visual signals. Yet, looming and receding sounds had a pronounced effect on the visual looming bias. Moreover, the significant interactions of sound direction with visual and auditory stimulus complexity reveals that the effect of looming/receding sound signals on the visual motion percept is enhanced for complex and perceptually immersive audiovisual stimulus pairs such as a visual Shepard image sequence and auditory Shepard tones. As participants were instructed to selectively report the percept in the visual modality and ignore the concurrent sound, we expect that looming sounds can influence the visual motion percept in the absence of attention. However, as it is difficult to control that participants exclusively focus on the visual inputs and ignore the sound, the increase in looming bias may to some extent also be caused by looming sounds in the presence of attention.

## Discussion

The longer dominance times for looming relative to receding percepts in binocular rivalry demonstrate that looming signals are prioritized in visual perception. Yet, the looming bias in visual perception is influenced by concurrent sounds: looming sounds amplify the visual looming bias, while receding sound decrease it and convert it into a receding bias. Critically, these audiovisual interactions depended on the statistical structure of the auditory and visual signals. The modulatory effects of looming and receding sounds on the visual looming bias were most pronounced when both the visual and auditory signals were structurally complex and perceptually immersive Shepard stimuli.

The looming bias during binocular rivalry reveals intimate links between the systems that regulate perception and action. It suggests that the perceptual access of sensory signals is optimized to maximize rewards and minimize losses of an organism's interactions with the environment [16], [18]. Thus, even if the task does not explicitly require any overt action as in our binocular rivalry experiments, looming stimuli that signal potential danger or collision are prioritized, so that an appropriate motor response such as freezing, fight or flight can be rapidly generated [1]–[3]. These perceptual biases may even be hardwired in the visual system as a consequence of selection pressures in the course of evolution.

In the natural environment, looming signals are encountered not only in the visual but also in the auditory modalities challenging the brain to integrate looming signals from all senses. Indeed, our results demonstrate profound audiovisual interactions across vision and audition in binocular rivalry. While auditory looming sounds amplify the visual looming bias, receding sounds reduce it and even convert it into a receding bias. Even though the size of the receding bias was smaller, it was observed robustly especially for complex audiovisual signals. Our results challenge previous studies [17] and suggest that audiovisual directional congruency can even override the natural looming bias of the visual system and convert it into a receding bias. Interestingly, these reliable audiovisual interactions were observed also, when simple auditory stimuli were combined with complex Shepard stimuli or complex Shepard sounds were presented together with simple visual stimuli. Thus, they even emerge for audiovisual signals that do not co-occur together in the natural environment, but are only linked in a metaphoric sense such as ‘illusionary’ pitch in Shepard sounds and radial motion in simple visual stimuli. Indeed, there is accumulating evidence that metaphoric (or synaesthetic) relationships play a critical role in multisensory binding [33], [34]. For instance, a recent study demonstrated that temporal ventriloquism is enhanced for synaesthetically congruent (i.e. auditory pitch, visual size) relative to incongruent stimuli [34].

The critical question of this study was whether the visual looming bias per se or the auditory influences on the looming bias depend on the statistical structure of the visual or auditory signals. While the visual looming bias per se was not influenced by the statistical structure of the audiovisual signals, the audiovisual interactions strongly depended on the nature of the audiovisual signals. The auditory influence on the visual looming bias was most pronounced when auditory and visual signals were complex Shepard stimuli that conformed more closely to the statistics of natural biologically relevant stimuli. Thus, the visual Shepard stimulus shared the characteristic 1/f amplitude spectrum with natural image statistics [32], [35]–[39]. The critical role of the signal structure for audiovisual interactions may be explained by several factors:

First, neurophysiological and behavioural evidence suggests that the visual processing system is tuned and optimized for natural image statistics [23], [24], [40], [41]. For instance, in binocular rivalry, noise images with an amplitude spectrum of 1/*f* have been shown to dominate over noise images with other spectral slopes suggesting that natural images are preferentially encoded and selected for perception [25]. Likewise, the auditory system exploits the statistics of natural sound sources such as broadband sounds or harmonic vocalizations [19]. Further, increased neural responses for looming stimuli in auditory cortex were observed only for complex sounds but not for white noise stimuli that violate the natural auditory input statistics [8]. Collectively, these findings suggest that the pronounced audiovisual interactions for Shepard stimuli may rely on neural mechanisms that are specialized for encoding more complex statistics of auditory and visual signals.

Second, from a cognitive perspective, the Shepard stimuli were perceived by participants as perceptually more immersive and ecologically valid than the simple looming and receding stimuli. For instance, subjects reported to perceive the visual and auditory Shepard stimuli as approaching clouds or rocks and alarming sounds. Many previous studies have revealed influences of higher cognitive factors such as attention or semantic congruency on binocular rivalry [40]–[42]. Likewise, multisensory integration depends on subjects' percept and cognitive set and is not determined by bottom-up sensory inputs alone. For instance, the classical McGurk illusion depends on whether subjects actively perceive both the visual and auditory inputs as speech [43]–[45]. Furthermore, when congruent looming sounds are presented together with stationary sounds, they amplify the looming bias in binocular rivalry primarily when they are attended. Collectively, these studies demonstrate that multisensory interactions may at least to some extent depend on attention. Conversely, the critical role of attention in perceptual selection as in binocular rivalry can be further enhanced via multisensory interactions [18]. In line with these findings, the perceptual immersiveness of the complex Shepard stimuli may enhance the interactions between auditory and visual looming signals via top-down modulatory mechanisms.

Third, over the past decade, multisensory interactions have been shown in widespread neural systems encompassing subcortical, primary sensory and association areas [46]–[49]. While audiovisual interactions in lower level sensory areas depended profoundly on spatiotemporal coincidence, they were influenced by subjects' percept and decisional processes in higher order association areas [50]–[52]. Thus, multisensory integration may emerge in a processing hierarchy with different types of information being integrated at different cortical levels. This hierarchical organization of multisensory integration then allows auditory and visual Shepard signals to interact at multiple cortical levels (for a review see [53]). In contrast, audiovisual interactions for simple random dot kinematograms or AM-FM tones may be confined to only a subset of these cortical areas.

In conclusion, visual perception prioritizes processing of biologically relevant looming stimuli especially when paired with looming auditory signals. Critically, these audiovisual interactions are amplified for statistically complex signals that engage neural processing at multiple levels of the cortical hierarchy and are perceptually more immersive.

## Supporting Information

Figure S1 **Visual stimulus characteristics.** Figure S1a shows the power spectral density of the Shepard stimulus (blue) and the starfield (green). Unlike the starfield stimulus, the Shepard stimulus shows the characteristic decrease in log power with increasing frequency resembling a 1/f curve. Figure S1b. The histograms show the absolute frequency of pixels in the visual Shepard and Starfield stimuli at each intensity value. While the distribution of intensity values in the Shepard stimulus is rather wide and thereby similar to natural images, it is narrow with a spike at a particular intensity value for the visual starfield.
(TIF)

Table S1 **Results of post-hoc t-tests for the non-motion tone (static sound) and no-sound control conditions comparing mean dominance durations (in sec) for looming vs. receding percepts.**
(DOC)
