# Supplementary figures and images for: Naturalistic Stimulus Structure Determines the Integration of Audiovisual Looming Signals in Binocular Rivalry
Source: PLoS One. 2013 Aug 27;8(8):e70710. doi: 10.1371/journal.pone.0070710 (PMC3754975; doi:10.1371/journal.pone.0070710)

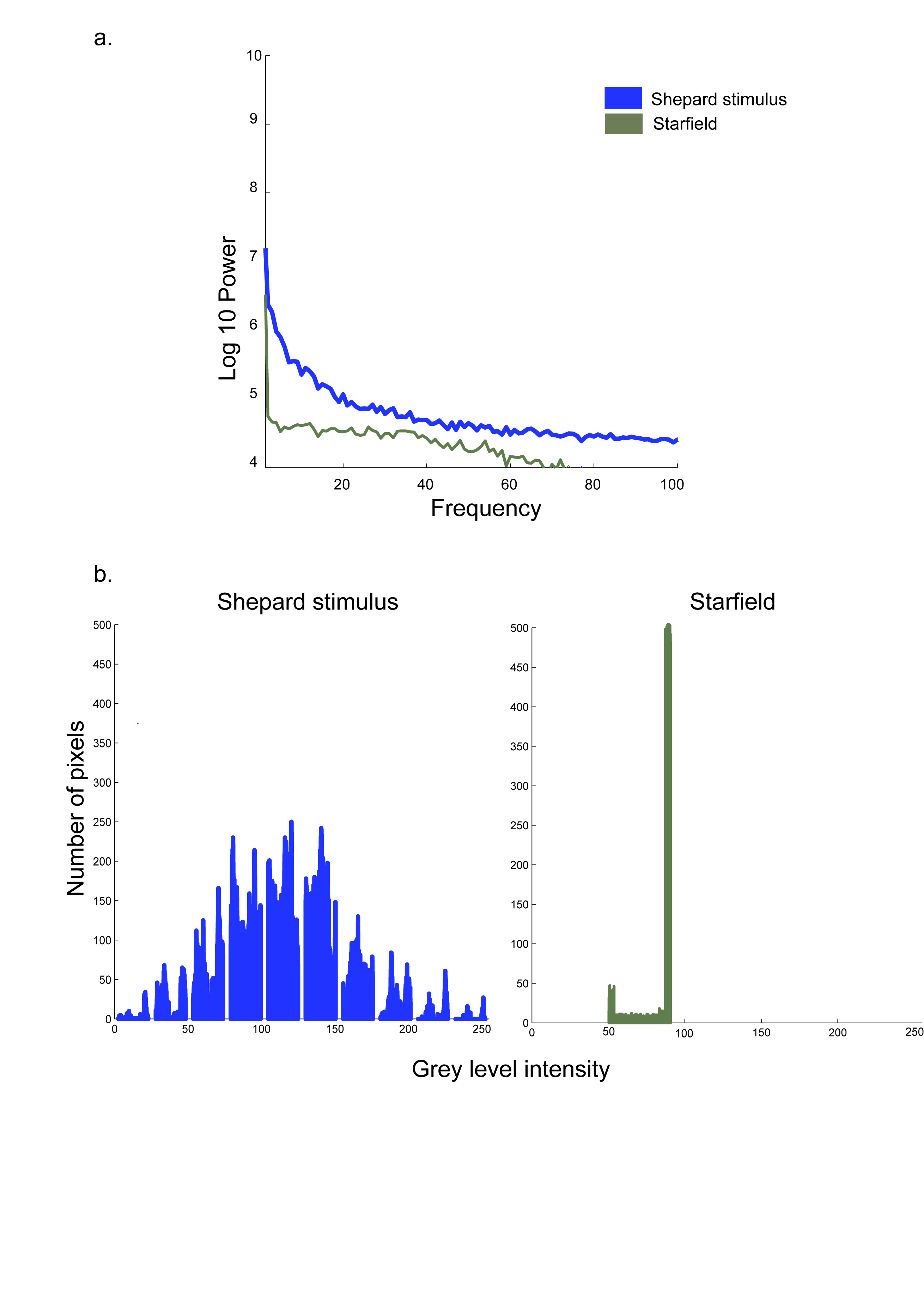

Supplement: Figure S1 — Visual stimulus characteristics. Figure S1a shows the power spectral density of the Shepard stimulus (blue) and the starfield (green). Unlike the starfield stimulus, the Shepard stimulus shows the characteristic decrease in log power with increasing frequency resembling a 1/f curve. Figure S1b. The histograms show the absolute frequency of pixels in the visual Shepard and Starfield stimuli at each intensity value. While the distribution of intensity values in the Shepard stimulus is rather wide and thereby similar to natural images, it is narrow with a spike at a particular intensity value for the visual starfield. (TIF) [file pone.0070710.s001.tif]
