# Supplementary material for: Naturalistic Stimulus Structure Determines the Integration of Audiovisual Looming Signals in Binocular Rivalry
Source: PLoS One. 2013 Aug 27;8(8):e70710. doi: 10.1371/journal.pone.0070710 (PMC3754975; doi:10.1371/journal.pone.0070710)
Supplement: Table S1 — Results of post-hoc t-tests for the non-motion tone (static sound) and no-sound control conditions comparing mean dominance durations (in sec) for looming vs. receding percepts. (DOC) [file pone.0070710.s002.doc]

| **Control Conditions** | Visual starfield | Visual Shepard |
| --- | --- | --- |
| Non-motion sound | t(15) = -0.85, p > 0.05 | t(15) = 0.84, p > 0.05 |
| No sound | t(15) = 1.13, p = 0.1 | t(15) = 2.52, p < 0.05 |
